# Technical and oncological safety of laparoscopic gastrectomy for gastric cancer in elderly patients ≥ 80 years old

**DOI:** 10.1186/s12877-022-03180-7

**Published:** 2022-06-02

**Authors:** Yoshitake Ueda, Norio Shiraishi, Hajime Fujishima, Takahide Kawasaki, Shigeo Ninomiya, Hidefumi Shiroshita, Tsuyoshi Etoh, Masafumi Inomata

**Affiliations:** 1 Department of Comprehensive Surgery for Community Medicine, Oita University Faculty of Medicine, Hasama-machi, Oita, 879-5593 Japan; 2 Department of Gastroenterological and Pediatric Surgery, Oita University Faculty of Medicine, Oita, Japan

**Keywords:** Gastric cancer, Elderly, Laparoscopic surgery, Safety, Curability

## Abstract

**Background:**

As the incidence of gastric cancer increases in elderly patients worldwide, laparoscopic gastrectomy (LG) for elderly patients with gastric cancer is also increasing. However, whether LG is an optimal surgical modality for elderly patients with gastric cancer remains unclear. This study aimed to evaluate the technical and oncological safety of LG for elderly patients ≥ 80 years old with gastric cancer.

**Methods:**

Patients who received curative gastrectomy for gastric cancer from 2003 to 2015 were enrolled in the study. They were divided into the LG in elderly patients aged over 80 years (LG-E) group, open gastrectomy (OG) in elderly patients (OG-E) group, and LG in non-elderly patients < 80 years (LG-NE) group. Patients’ demographics and short- and long-term outcomes, such as postoperative complications and 5-year survival rate, were compared between the three groups, retrospectively.

**Results:**

The LG-E, OG-E, and LG-NE groups comprised 45, 43, and 329 patients, respectively. In the comparison between the LG-E and OG-E groups, the incidence of distal gastrectomy (DG) and the proportions of patients with pathological tumor stage T1, pathological N0, and final stage I were significantly higher in the LG-E versus OG-E group (89 vs. 56%, 76% vs. 16%, 82% vs. 37%, and 84% vs. 35%, *p* < 0.01, respectively). Blood loss and the incidence of overall postoperative complications in the LG-E group were significantly lower than those in the OG-E group (40 vs. 240 g, *p* < 0.01, and 29% vs. 53%, *p* < 0.05, respectively). Although the 5-year overall survival (OS) rate was not significantly different between the two groups, the 5-year disease-specific survival (DSS) rate was significantly higher in the LG-E group versus OG-E group (93% vs. 78%, *p* < 0.05). Overall comorbidities were significantly higher in the LG-E group versus LG-NE group, but there were no significant differences in short-term outcomes between the two groups. Further, although the 5-year OS rate was significantly lower in the LG-E group versus LG-NE group (67% vs. 87%, *p* < 0.01), there was no significant difference between the two groups in 5-year DSS rate.

**Conclusion:**

LG is technically and oncologically safe for the treatment of gastric cancer in both elderly patients aged ≥ 80 years and the non-elderly and can be an optimal surgical modality for elderly patients with gastric cancer.

## Background

Gastric cancer is the 6th most common cancer type and the third leading cause of death among all malignancies worldwide [1]. In Japan, gastric cancer was ranked as the second most common cancer type and the third leading cause of cancer-related deaths in 2019 [2]. In addition, Japan is faced with an unprecedented ageing society due to the extension of life expectancy. According to the World Health Statistics published by World Health Organization in 2021, the average life span of the Japanese is 84.3 years, which is the longest life expectancy in the world for both men and women [3]. Consequently, the incidence of gastric cancer in elderly patients has been increasing year by year in Japan, with more than 30% of gastric cancer patients now aged 80 years or over [4].

Laparoscopic gastrectomy (LG) for the treatment of gastric cancer has been widely performed throughout the world because of its advantages, such as less damage to the patients, faster recovery of digestive function, and shorter hospital stay compared with open gastrectomy (OG) [5–8]. Many previous studies including large-sample, multicenter, and randomized controlled trials have demonstrated these clinical benefits of LG for gastric cancer [9–12]. Thus, LG has been regarded as a standard procedure for early gastric cancer, and the indication for LG is expanding to advanced gastric cancer [13–15]. Then, LG has also been rapidly used in elderly patients aged 80 years or older with gastric cancer in Japan. However, these previous clinical trials of LG were conducted on patients excluded the elderly patients aged 80 years or older, because it is often considered that elderly patients have high risk factors for gastrectomy due to decreased organ function and many comorbidities. Therefore, technical and oncological safety of LG for gastric cancer in elderly patients ≥ 80 years old is still unclear.

This study aimed to evaluate the technical and oncological safety of LG for elderly patients ≥ 80 years old with gastric cancer, and to clarify whether LG is an optimal procedure for elderly patients with gastric cancer.

## Patients and methods

### Patients

Four-hundred seventeen patients with gastric cancer who had undergone curative surgery in our department between January 2003 and December 2015 were enrolled in this study. All patients judged by the anesthesiologist to be operable were indicated for surgery. Patients who had undergone palliative and emergent operations were excluded (*n *= 39, 9%). All patients were classified into three groups. The LG in elderly patients (LG-E) group included 45 patients aged ≥ 80 years who had undergone LG. The OG in elderly patients (OG-E) group included 43 patients aged ≥ 80 years who had undergone OG. The LG in non-elderly patients (LG-NE) group included 329 patients aged < 80 years who had undergone LG.

### Methods

Patients’ demographics, preoperative and operative variables, clinicopathological findings, and postoperative short- and long-term outcomes for all patients were obtained from the patients’ medical records, operation records, and pathology records in our hospital database. The three groups were examined and compared in terms of patient characteristics such as age, sex, body mass index, presence of symptoms, previous endoscopic mucosal dissection (ESD), previous abdominal surgery, comorbidities, and pathological findings including tumor location, tumor differentiation, tumor size, pTNM staging as determined on the basis of the 14^th^ edition of the Japanese Classification of Gastric Carcinoma [16], and short-term outcomes including operation time, blood loss, intraoperative complication, days to solid diet, length of hospital stay, and postoperative complications. Postoperative mortality was defined as death within 30 days of operation. Postoperative complications included anastomotic leakage, ileus, enterocolitis, intra-abdominal abscess, delayed gastric emptying, and pneumonia were defined as any condition requiring conservative or surgical treatment occurring within 30 days after operation. Postoperative complications were assessed using the Clavien-Dindo classification (CD) categories [17]. From the pathological records, the depth of invasion was examined at the longest cut section line of the tumor, and lymph node metastasis was examined at the largest cut section of the lymph node. All tissues were examined by expert pathologists. Long-term outcomes were compared between each group in terms of 5-year overall survival (OS) and 5-year disease-specific survival (DSS). Patients were routinely followed up every 6 months during the first 5 years. OS was defined as the period between the date of operation to the date of death for any cause or the end of follow-up, and DSS was defined as the period between the date of operation and the date of death due to primary gastric cancer or the end of follow-up.

This study was approved by the Ethical Committee of Oita University Faculty of Medicine (Approval No. 542), and all patients included in the study gave their written informed consent.

### Statistical analysis

Quantitative data are given as the median and range. Differences between the three groups were assessed by the chi-square test, Fisher’s exact test, or Mann–Whitney U test as appropriate. Long-term outcomes were compared between each group by log-rank test and are summarized as Kaplan–Meier curves and hazards ratios with 95% confidence intervals. A *p*-value of < 0.05 was considered to indicate statistical significance. These analyses were carried out using SPSS ver. 24 (SPSS Inc., Chicago, IL, USA).

## Results

Characteristics of the patients in the three groups are given in Table 1. The average age of the patients in the LG-E, OG-E, and LG-NE groups were 84, 84, and 64 years, respectively. The frequency of patients with preoperative symptoms was lower in the LG-E group versus OG-E group (42% vs. 63%, *p* < 0.05). The LG-E group had a higher percentage of patients with previous ESD than the OG-E and LG-NE groups (24% vs. 2% and 12%, *p* < 0.01 and *p* < 0.05, respectively). The incidence of overall comorbidities was significantly higher in the LG-E group versus LG-NE group (67% vs. 48%, *p* < 0.05). Total gastrectomy was performed less frequently in the LG-E group than in the OG-E group (4% vs. 44%, *p* < 0.01).

**Table 1:** Patient characteristics

Factors	LG-E group	OG-E group	LG-NE group	LG-E vs OG-E	LG-E vs LG-NE
	**(*n* = 45)**	**(*n* = 43)**	**(*n* = 329)**	***P*-value**	***P*-value**
Age (years, mean ± SD)	84 ± 3	84 ± 3	64 ± 11	NS	** < 0.01**
Gender					
Male	34 (76%)	30 (70%)	220 (67%)	NS	NS
Female	11 (24%)	13 (30%)	109 (33%)		
Body mass index	22 ± 3	21 ± 3	23 ± 3	NS	NS
Presence of symptom	19 (42%)	27 (63%)	141 (43%)	** < 0.05**	NS
Previous endoscopic submucosal dissection	11 (24%)	1 ( 2%)	39 (12%)	** < 0.01**	** < 0.05**
Previous abdominal surgery	11 (24%)	8 (19%)	98 (30%)	NS	NS
Comorbidities					
Overall comorbidity	30 (67%)	30 (70%)	158 (48%)	NS	** < 0.05**
Cardiac disease	8 (18%)	10 (23%)	39 (12%)	NS	NS
Hypertension	15 (33%)	9 (21%)	57 (17%)	NS	** < 0.05**
Diabetes mellitus	4 (9%)	3 (7%)	34 (10%)	NS	NS
Respiratory disease	5 (11%)	9 (21%)	15 (5%)	NS	NS (0.08)
Renal disease	2 (4%)	1 (2%)	4 (1%)	NS	NS
Cerebrovascular disease	3 (7%)	6 (14%)	23 (7%)	NS	NS
Operative method DG/PG/TG	40 (89%)/3 (7%)/2 (4%)	24 (56%)/0/19 (44%)	277 (84%)/28 (9%)/24 (7%)	** < 0.01**	NS

*LG-E* Laparoscopic gastrectomy in elderly patients, *OG-E* Open gastrectomy in elderly patients, *LG-NE* Laparoscopic gastrectomy in non-elderly patients, *SD* Standard deviation, *NS* Not significant, *DG* Distal gastrectomy, *PG* Proximal gastrectomy, *TG* Total gastrectomy

**Fig. 1:**
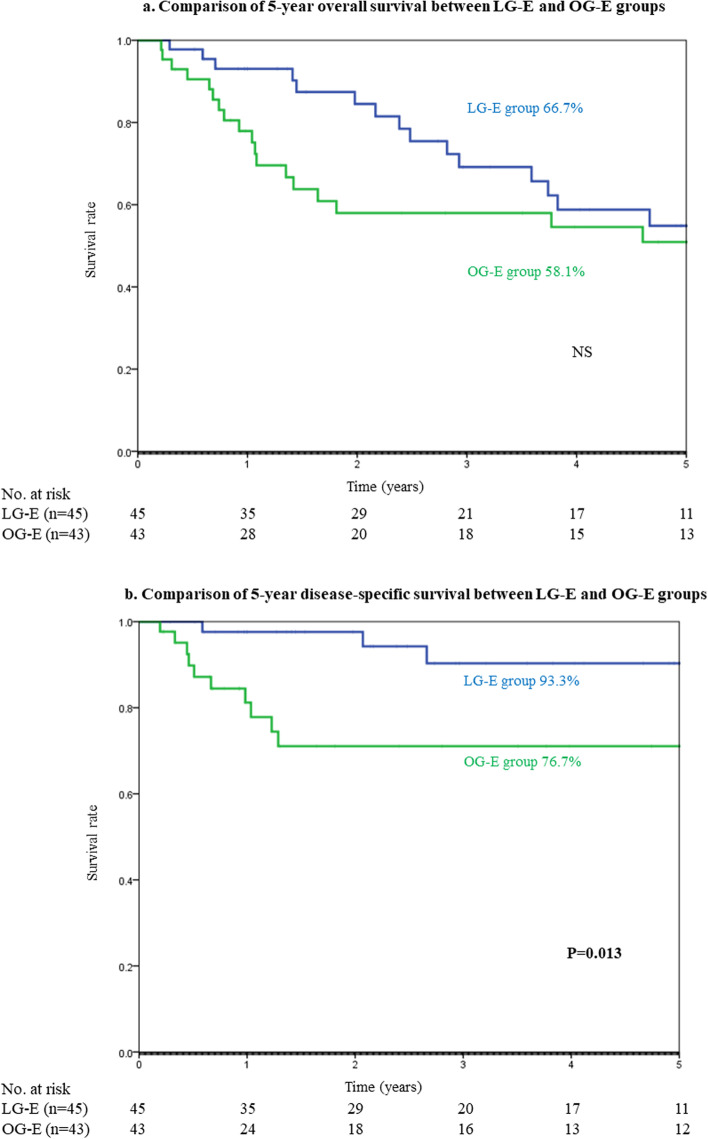
**a** Comparison of 5-year overall survival between the LG-E and OG-E groups. **b** Comparison of 5-year disease-specific survival between the LG-E and OG-E groups. *LG-E* laparoscopic gastrectomy in elderly patients, *OG-E* open gastrectomy in elderly patients

Pathological findings and short-term outcomes can be compared between the LG-E and OG-E groups in Table 2. Regarding tumor location, the frequency of upper gastric cancer was lower in the LG-E group versus OG-E group (13% vs. 35%, *p* < 0.05). The median tumor size in the LG-E group was smaller than that in the OG-E group (36 vs. 69 mm, *p* < 0.01). The proportions of patients who had pathological tumor stage T1, pathological N0, and final stage I were significantly higher in the LG-E group versus OG-E group (76% vs. 16%, 82% vs. 37%, and 84% vs. 35%, *p* < 0.01, respectively). Regarding the short-term outcomes, the amount of blood loss in the LG-E group was significantly lower than that in the OG-E group (40 vs. 240 g, *p* < 0.01). However, there were no significant differences in operation time, the incidence of intraoperative complications, and days to solid diet between the two groups. The incidence of overall postoperative complications in the LG-E group was significantly lower than that in the OG-E group (29% vs. 53%, *p* < 0.05), and the length of hospital stay in the LG-E group was shorter than that in the OG-E group (19 vs. 24 days, *p* < 0.05). In the analysis of long-term outcomes, although there was no significant difference between the two groups in 5-year OS rate (67% vs. 58%) (Fig. 1a), the 5-year DSS rate in the LG-E group was significantly higher than that in the OG-E group (93% vs. 78%, *p* < 0.05) (Fig. 1b).

**Table 2:** Pathological findings and short-term outcomes in the LG-E and OG-E groups

Factors	LG-E group	OG-E group	*P*-value
	(*n* = 45)	(*n* = 43)	
**Pathological findings**			
Tumor location			
Upper	6 (13%)	15 (35%)	**< .05**
Middle	19 (42%)	11 (26%)	
Lower	20 (44%)	17 (40%)	
Tumor differentiation			
Well/moderately	29 (64%)	20 (47%)	NS (0.07)
Poorly/mucinous	16 (36%)	23 (53%)	
Tumor size (mm, mean ± SD)	36 ± 20	69 ± 37	**< .01**
pT stage			
T1	34 (76%)	7 (16%)	**< .01**
T2-T4	11 (24%)	36 (84%)	
pN stage			
N0	37 (82%)	16 (37%)	**< .01**
N1-N2	8 (18%)	27 (63%)	
TNM Stage			
I	38 (84%)	15 (35%)	**< .01**
II-IV	7 (16%)	28 (65%)	
**Short-term outcomes**			
Operation time (min, mean ± SD)	288 ± 84	258 ± 73	NS (0.08)
Blood loss (g, mean ± SD)	86 ± 162	433 ± 515	**< .01**
Lymph node dissection			
< D2	38 (84%)	20 (47%)	** < .01**
D2	7 (16%)	23 (53%)	
Intraoperative complication	0	0	NS
Days to solid diet (days, mean ± SD)	5.6 ± 5.2	5.4 ± 2.4	NS
Length of hospital stay (days, mean ± SD)	19 ± 9	24 ± 13	**< .05**
Postoperative complication			
Mortality	0	0	NS
Overall morbidity	13 (29%)	23 (53%)	**< .05**
Overall morbidity (CD grade 3 or more)	2 ( 4%)	6 (14%)	NS
Anastomotic leakage	1 (2%)	4 (9%)	NS
Ileus	0	1 (2%)	NS
Enterocolitis	2 (4%)	3 (7%)	NS
Intraabdominal abscess	0	1 (2%)	NS
Delayed gastric emptying	4 (9%)	7 (16%)	NS
Pneumonia	3 (7%)	6 (14%)	NS
Others	4 (9%)	3 (7%)	NS

*LG-E* Laparoscopic gastrectomy in elderly patients, *OG-E* Open gastrectomy in elderly patients, *NS* Not significant, *SD* Standard deviation, *CD* Clavien-Dindo

**Fig. 2:**
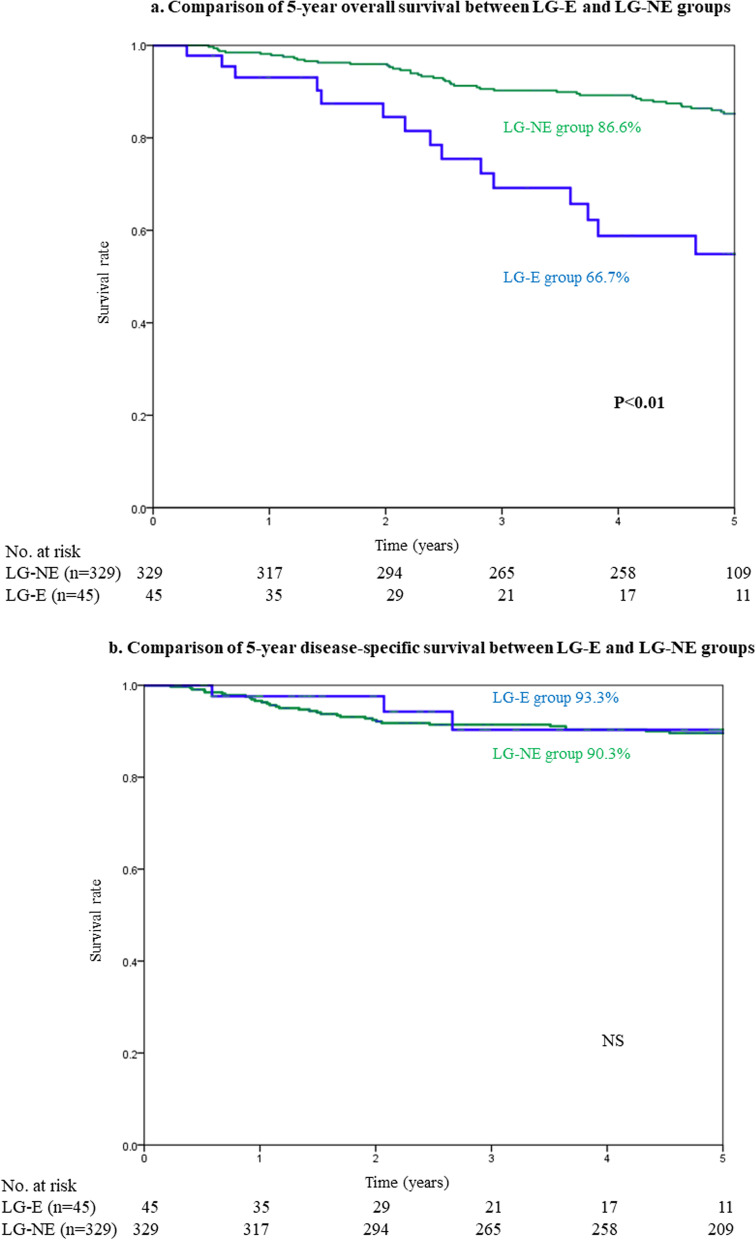
**a** Comparison of 5-year overall survival between the LG-E and LG-NE groups. **b** Comparison of 5-year disease-specific survival between the LG-E and LG-NE groups. *LG-E* laparoscopic gastrectomy in elderly patients, *LG-NE* laparoscopic gastrectomy in non-elderly patients

Pathological findings and short-term outcomes can be compared between the LG-E and LG-NE groups in Table 3. There were no significant differences in the pathological findings between the two groups. Among the short-term outcomes, there were no significant differences in operative methods, operation time, blood loss, the incidence of intraoperative complications, days to solid diet, length of hospital stay, and the incidence of postoperative complications between the two groups. Regarding the long-term outcomes, the 5-year OS rate in the LG-E group was significantly lower than that in the LG-NE group (67% vs. 87%, *p* < 0.01) (Fig. 2a), but the difference between the two groups in 5-year DSS (93% vs. 90%) was not significant (Fig. 2b).

**Table 3:** Pathological findings and short-term outcomes in the LG-E and LG-NE groups

Factors	LG-E group	LG-NE group	*P*-value
	(*n* = 45)	(*n* = 329)	
**Pathological findings**			
Tumor location			
Upper	6 (13%)	62 (19%)	NS
Middle	19 (42%)	136 (41%)	
Lower	20 (44%)	131 (40%)	
Tumor differentiation			
Well/moderately	29 (64%)	174 (53%)	NS
Poorly/mucinous	16 (36%)	155 (47%)	
Tumor size (mm, mean ± SD)	36 ± 20	31 ± 21	NS
pT stage			
T1	34 (76%)	239 (83%)	NS
T2-T4	11 (24%)	90 (27%)	
pN stage			
N0	37 (82%)	265 (81%)	NS
N1-N2	8 (18%)	64 (19%)	
TNM Stage			
I	38 (84%)	271 (82%)	NS
II-IV	7 (16%)	58 (18%)	
**Short-term outcomes**			
Operation time (min, mean ± SD)	288 ± 84	306 ± 77	NS
Blood loss (g, mean ± SD)	86 ± 162	102 ± 217	NS
Lymph node dissection			
< D2	38 (84%)	256 (78%)	NS
D2	7 (16%)	73 (22%)	
Postoperative complication			
Mortality	0	0	NS
Overall morbidity	13 (29%)	78 (24%)	NS
Overall morbidity (CD grade 3 or more)	2 ( 4%)	26 (8%)	NS
Anastomotic leakage	1 (2%)	5 (2%)	NS
Ileus	0	1 (0.3%)	NS
Enterocolitis	2 (4%)	2 (0.6%)	NS (0.07)
Intraabdominal abscess	0	4 (1%)	NS
Delayed gastric emptying	4 (9%)	38 (12%)	NS
Pneumonia	3 (7%)	8 (2%)	NS
Others	4 (9%)	17 (5%)	NS

*LG-E* Laparoscopic gastrectomy in elderly patients, *LG-NE* Laparoscopic gastrectomy in non-elderly patients, *NS* Not significant, *SD* Standard deviation, *CD* Clavien-Dindo

## Discussion

In the present study, the blood loss and the incidence of postoperative complications were significantly lower in the LG group than OG group in the elderly patients. The 5-year DSS rate in the LG group was better than that in the OG group because of the higher frequency of patients with advanced gastric cancer in the OG group. In comparison between the elderly and non-elderly LG groups, there were no significant differences in the pathological findings and short-term outcomes. Further, although the 5-year OS rate in the elderly group was worse than that in the non-elderly group, there was no significant difference between the two groups in 5-year DSS. These results showed that LG for elderly patients with gastric cancer is technically safe and less invasive than OG and is also oncologically safe, same as non-elderly patients.

For elderly patients undergoing LG, it is generally considered that special attention must be paid because of their reduced organ function and increased co-morbidities, such as cardiac, pulmonary, and renal diseases. However, some studies suggested that LG for elderly patients with gastric cancer offers several clinical advantages over OG, as in non-elderly patients. Honda et al. reported in the first and largest prospective cohort study conducted in Japan that LG shortened the length of the postoperative hospital stay more than did OG in elderly patients with gastric cancer [18]. Tanaka et al. described that LG was safe and had some advantages such as lower complication rate and faster recovery than OG in propensity-matched patients aged over 80 years [19]. In the present study investigating the advantages and disadvantages of LG compared to OG in the elderly, we also showed that short-term outcomes including blood loss and the incidence of postoperative complications were better in the LG group than those in the OG group. It is general knowledge that a reduction in intraoperative blood loss leads to a decrease in postoperative complications [20]. Consequently, although we haven’t introduced an enhanced recovery after surgery protocol in our department yet, the length of hospital stay in LG group was also shorter than that in the OG group, which suggests that LG in elderly patients is not only safe but also less invasive. We believe that LG for elderly patients is a useful surgical procedure that can reduce postoperative complications.

Gastrectomy for elderly patients with gastric cancer is remarkably associated with a higher incidence of postoperative pneumonia, which can lead to lowering of the quality of life and postoperative death [21, 22]. Therefore, many surgeons are concerned that elderly patients have a limited capacity to tolerate gastrectomy. As well, more attention has been paid in recent years to preoperative evaluation of conditions such as sarcopenia and frailty in elderly patients [19, 23]. Kim and Kim used propensity score matching to investigate the outcomes of LG in very elderly gastric cancer patients whose age exceeded the average lifespan of the Korean population [24]. They reported that only pulmonary complications were more frequent in this elderly group. In their meta-analysis, Pan et al. also showed that elderly patients with gastric cancer were associated with a higher rate of pulmonary complications following LG [25]. We also investigated whether there were any postoperative complications peculiar to the elderly undergoing LG by comparison between the LG-E and LG-NE groups. The proportion of overall comorbidities was higher in the LG-E group than that in the LG-NE group. However, there were no significant differences in the incidences of postoperative complications directly attributable to poor functional capacity, such as postoperative pneumonia, between the two groups. Other authors showed similar results. Komori et al. reported that the short-term outcomes after gastrectomy without regard to approach were almost equal between non-elderly and elderly patients [26]. Mikami et al. also showed that there were no differences in short-term outcomes including postoperative morbidity between elderly and non-elderly patients who underwent LG [27]. Our results showed that the rate of postoperative complications in the elderly patients did not increase compared with that in the non-elderly patients, despite the higher incidence of comorbidities in the elderly patients. We believe that LG is more suitable for elderly patients with gastric cancer because LG helps to prevent postoperative pulmonary complications.

We also investigated whether LG would worsen the prognosis in the elderly. Our study showed that although the 5-year OS rate in the elderly group was worse than that in the non-elderly group, there was no significant difference in 5-year DSS rate between the two groups. There is little evidence on the long-term outcomes of LG compared to short-term outcomes in the elderly gastric cancer patients. Some studies reported that long-term outcomes of elderly patients in a laparoscopic group were similar to those for non-elderly patients [28, 29]. Shimada et al. showed that although 5-year OS was significant lower in the elderly group than in the non-elderly group, 5-year DSS was similar in the two groups, as with our results [30]. Ushimaru et al. reported that although DSS was similar between the laparoscopic and open groups among young and elderly patients, the laparoscopic group was associated with more favorable OS than the open group only among the elderly patients because of the lower number of deaths from respiratory diseases [31]. Some reports indicated that the incidence of postoperative complications was an important factor that influenced long-term outcomes [24, 32–34]. In the present study, the incidence of postoperative complications after LG was equal between the elderly and non-elderly patients. Therefore, we consider that LG without severe postoperative complications tends to lead to a good prognosis. To ensure favorable long-term outcomes in elderly patients after LG, surgeons need to carefully perform both the operation and perioperative intensive care to prevent postoperative complications.

There are some limitations in this study. First, this retrospective study was conducted in a single low-volume center. Second, because the patients weren’t randomized, there was selection bias in regard to the choice of the operation method in this study because OG tended to be selected for more advanced gastric cancer patients. It was difficult to apply case-matching due to the insufficient sample size in this study. Third, we could not evaluate postoperative delirium, nutritional status, and quality of life after gastrectomy because of the retrospective design. In the future, a larger-sized prospective cohort study will be necessary.

In conclusion, LG for elderly patients aged ≥ 80 years with gastric cancer was technically safer and less invasive than OG and provided acceptable oncologic outcomes compared with non-elderly patients. Although there was selection bias, our results suggest that LG can be an optimal surgical modality for elderly patients aged ≥ 80 years with gastric cancer. To confirm our findings, a multi-center prospective study with a larger sample size will be required in the future.

## Data Availability

All data generated or analyzed during this study are included in this published article.

## Abbreviations

DSS: Disease-specific survival
ESD: Endoscopic mucosal dissection
LG: Laparoscopic gastrectomy
LG-E: LG in elderly patients
LG-NE: LG in non-elderly patients
OG: Open gastrectomy
OG-E: OG on elderly patients
OS: Overall survival

## References

[1] Sung H, Ferlay J, Siegel LR, Laversanne M, Soerjomataram I, Jemal A (2021). Global Cancer Statistics 2020: GLOBOCAN estimates of incidence and mortality worldwide for 36 cancers in 185 countries. CA Cancer J Clin.

[2] Center for Cancer Control and Information Services. 2021. Cancer Statistics in Japan. National Cancer Center, Japan. https://ganjoho.jp/reg_stat/statistics/stat/summary.html. Accessed 5 Jan 2022. In Japanese.

[3] The Global Health Observatory. World Health Organization. https://www.who.int/data/gho/data/indicators/indicator-details/GHO/life-expectancy-at-birth-(years). Accessed 5 Jan 2022.

[4] Cancer Registry and Statistics. (Monitoring of Cancer Incidence in Japan (MCIJ)). Cancer Information Service, National Cancer Center, Japan. https://ganjoho.jp/reg_stat/statistics/dl/index.html. Accessed 5 Jan 2022. In Japanese.

[5] Katai H, Mizusawa J, Katayama H, Takagi M, Yoshikawa T, Fukagawa T (2017). Short-term surgical outcomes from a phase III study of laparoscopy-assisted versus open distal gastrectomy with nodal dissection for clinical stage IA/IB gastric cancer: Japan Clinical Oncology Group Study JCOG0912. Gastric Cancer.

[6] Huscher CG, Mingoli A, Sgarzini G, Sansonetti A, Di Paola M, Recher A (2005). Laparoscopic versus open subtotal gastrectomy for distal gastric cancer: five year results of a randomized prospective trial. Ann Surg.

[7] Lee SI, Choi YS, Park DJ, Kim HH, Yang HK, Kim MC (2006). Comparative study of laparoscopy-assisted distal gastrectomy and open distal gastrectomy. J Am Coll Surg.

[8] Ohtani H, Tamamori Y, Noguchi K, Azuma T, Fujimoto S, Oba H (2010). A meta-analysis of randomized controlled trials that compared laparoscopy assisted and open distal gastrectomy for early gastric cancer. J Gastrointest Surg.

[9] Kurokawa Y, Katai H, Fukuda H, Sasako M, Gastric Cancer Surgical Study Group of the Japan Clinical Oncology Group (2008). Phase II study of laparoscopy-assisted distal gastrectomy with nodal dissection for clinical stage I gastric cancer: Japan Clinical Oncology Group Study JCOG0703. Jpn J Clin Oncol.

[10] Katai H, Sasako M, Fukuda H, Nakamura K, Hiki N, Saka M (2010). Safety and feasibility of laparoscopy-assisted distal gastrectomy with suprapancreatic nodal dissection for clinical stage I gastric cancer: a multicenter phase II trial (JCOG 0703). Gastric Cancer.

[11] Nakamura K, Katai H, Mizusawa J, Yoshikawa T, Ando M, Terashima M (2013). A phase III study of laparoscopy-assisted versus open distal gastrectomy with nodal dissection for clinical stage IA/IB gastric cancer (JCOG0912). Jpn J Clin Oncol.

[12] Kim W, Kim HH, Han SU, Kim MC, Hyung WJ, Ryu SW (2016). Decreased morbidity of laparoscopic distal gastrectomy compared with open distal gastrectomy for stage I gastric cancer: short-term outcomes from a multicenter randomized controlled trial (KLASS-01). Ann Surg.

[13] Hu Y, Huang C, Sun Y, Su X, Cao H, Hu J (2016). Morbidity and mortality of laparoscopic versus open D2 distal gastrectomy for advanced gastric cancer: a randomized controlled trial. J Clin Oncol.

[14] Shi Y, Xu X, Zhao Y, Qian F, Tang B, Hao Y (2018). Short-term surgical outcomes of a randomized controlled trial comparing laparoscopic versus open gastrectomy with D2 lymph node dissection for advanced gastric cancer. Surg Endosc.

[15] Wang Z, Xing J, Cai J, Zhang Z, Li F, Zhang N (2019). Short-term surgical outcomes of laparoscopy-assisted versus open D2 distal gastrectomy for locally advanced gastric cancer in North China: a multicenter randomized controlled trial. Surg Endosc.

[16] Japanese Gastric Cancer Association (2011). Japanese classification of gastric carcinoma: 3rd English edition. Gastric Cancer.

[17] Clavien PA, Barkun J, de Oliveira ML, Vauthey JN, Dindo D, Schulick RD (2010). The Clavien-Dindo classification of surgical complications: five-year experience. Ann Surg.

[18] Honda M, Kumamaru H, Etoh T, Miyata H, Yamashita Y, Yoshida K (2019). Surgical risk and benefits of laparoscopic surgery for elderly patients with gastric cancer: a multicenter prospective cohort study. Gastric Cancer.

[19] Tanaka T, Suda K, Inaba K, Umeki Y, Gotoh A, Ishida Y (2019). Impact of frailty on postoperative outcomes for laparoscopic gastrectomy in patients older than 80 years. Ann Surg Oncol.

[20] Huang CM, Tu RH, Lin JX, Zheng CH, Li P, Xie JW (2015). A scoring system to predict the risk of postoperative complications after laparoscopic gastrectomy for gastric cancer based on a large-scale retrospective study. Medicine (Baltimore).

[21] Yamada H, Shinohara T, Takeshita M, Umesaki T, Fujimori Y, Yamagishi K (2013). Postoperative complications in the oldest old gastric cancer patients. Int J Surg.

[22] Takeuchi D, Koide N, Suzuki A, Ishizone S, Shimizu F, Tsuchiya T (2015). Postoperative complications in elderly patients with gastric cancer. J Surg Res.

[23] Shen Y, Hao Q, Zhou J, Dong B (2017). The impact of frailty and sarcopenia on postoperative outcomes in older patients undergoing gastrectomy surgery: a systematic review and meta-analysis. BMC Geriatr.

[24] Kim DJ, Kim W (2018). Role of laparoscopic gastrectomy in very elderly patients with gastric cancer who have outlived the average lifespan. J Gastric Cancer.

[25] Pan Y, Chen K, Yu WH, Maher H, Wang SH, Zhao HF (2018). Laparoscopic gastrectomy for elderly patients with gastric cancer: a systematic review with meta-analysis. Medicine (Baltimore).

[26] Komori K, Kano K, Aoyama T, Hashimoto I, Hara K, Murakawa M (2020). The short- and long-term outcomes of gastrectomy in elderly patients with gastric cancer. In Vivo.

[27] Mikami R, Tanaka E, Murakami T, Ishida S, Matsui Y, Horita K (2021). The safety and feasibility of laparoscopic gastrectomy for gastric cancer in very elderly patients: short-and long-term outcomes. Surg Today.

[28] Yang XW, Zhu SH, Li PZ, Li WZ, Sun XL (2018). Outcomes of laparoscopic gastrectomy for gastric cancer in elderly patients. J BUON.

[29] Xu K, Xing J, Fan Y, Cui M, Zhang C, Yang H (2021). Effects of laparoscopic-assisted gastrectomy on elderly patients with gastric cancer. J BUON.

[30] Shimada S, Sawada N, Oae S, Seki J, Takano Y, Ishiyama Y (2018). Safety and curability of laparoscopic gastrectomy in elderly patients with gastric cancer. Surg Endosc.

[31] Ushimaru Y, Kurokawa Y, Takahashi T, Saito T, Yamashita K, Tanaka K (2020). Is laparoscopic gastrectomy more advantageous for elderly patients than for young patients with resectable advanced gastric cancer?. World J Surg.

[32] Saito T, Kurokawa Y, Miyazaki Y, Makino T, Takahashi T, Yamasaki M (2015). Which is a more reliable indicator of survival after gastric cancer surgery: Post-operative complication occurrence or C-reactive protein elevation?. J Surg Oncol.

[33] Tokunaga M, Tanizawa Y, Bando E, Kawamura T, Terashima M (2013). Poor survival rate in patients with postoperative intra-abdominal infectious complications following curative gastrectomy for gastric cancer. Ann Surg Oncol.

[34] Jiang N, Deng JY, Ding XW, Zhang L, Liu HG, Liang YX (2014). Effect of complication grade on survival following curative gastrectomy for carcinoma. World J Gastroenterol.

